# Regulation of heat shock proteins 70 and their role in plant immunity

**DOI:** 10.1093/jxb/erab549

**Published:** 2022-01-12

**Authors:** Miroslav Berka, Romana Kopecká, Veronika Berková, Břetislav Brzobohatý, Martin Černý

**Affiliations:** 1 Department of Molecular Biology and Radiobiology, Faculty of AgriSciences, Mendel University in Brno, CZ-61300 Brno, Czech Republic; 2 The James Hutton Institute, UK

**Keywords:** Biotic interactions, cytokinin, HSP70, immunity, plant defense, phytohormone

## Abstract

Heat shock proteins 70 (HSP70s) are steadily gaining more attention in the field of plant biotic interactions. Though their regulation and activity in plants are much less well characterized than are those of their counterparts in mammals, accumulating evidence indicates that the role of HSP70-mediated defense mechanisms in plant cells is indispensable. In this review, we summarize current knowledge of HSP70 post-translational control in plants. We comment on the phytohormonal regulation of *HSP70* expression and protein abundance, and identify a prominent role for cytokinin in HSP70 control. We outline HSP70s’ subcellular localizations, chaperone activity, and chaperone-mediated protein degradation. We focus on the role of HSP70s in plant pathogen-associated molecular pattern-triggered immunity and effector-triggered immunity, and discuss the contribution of different HSP70 subfamilies to plant defense against pathogens.

## Teaser

The extensive regulation of HSP70 has received little attention in plant science. Here, we summarize current knowledge gained in large-scale profiling experiments and highlight HSP70s’ role in plant immunity.

## Introduction

Heat shock proteins (HSPs) were discovered by Italian scientist Ferruccio Ritossa who noticed a rapid transcriptional response to heat shock in *Drosophila melanogaster* and eventually published his results in 1962 (see Ritossa, 1996). The term ‘heat shock proteins’ was adopted 12 years later (Tissiéres *et al.*, 1974). What are HSPs? These ubiquitous and widespread proteins across prokaryotic and eukaryotic organisms were first discovered in response to an increase in temperature, but accumulated evidence indicates that these proteins are involved in much more than that. Foremost, HSPs act as chaperones and are involved in the assembly, stabilization, and maturation of proteins and protein complexes (Wang *et al.*, 2004). Besides their chaperone functions, HSPs play a role in plant development and response to abiotic and biotic stress conditions, including drought, salinity, pathogen infection, and insect attacks (Park and Seo, 2015; Usman *et al.*, 2017; Ul Haq *et al.*, 2019). The HSP family covers a variety of proteins, ranging from 10 kDa to >100 kDa, and the molecular weight is the basis of present-day HSP nomenclature (Ratheesh Kumar *et al.*, 2012). Several recent publications have reviewed the role of plant HSP90 (Tichá *et al.*, 2020), HSP40 (Verma *et al.*, 2019), and small HSPs (Asea *et al*., 2016; Waters and Vierling, 2020). Here, we will outline the role of HSP70s, one of the most studied HSPs in animals, and discuss their somewhat overlooked roles in plants.

### HSP70 structure is evolutionarily conserved

**Fig. 1.**
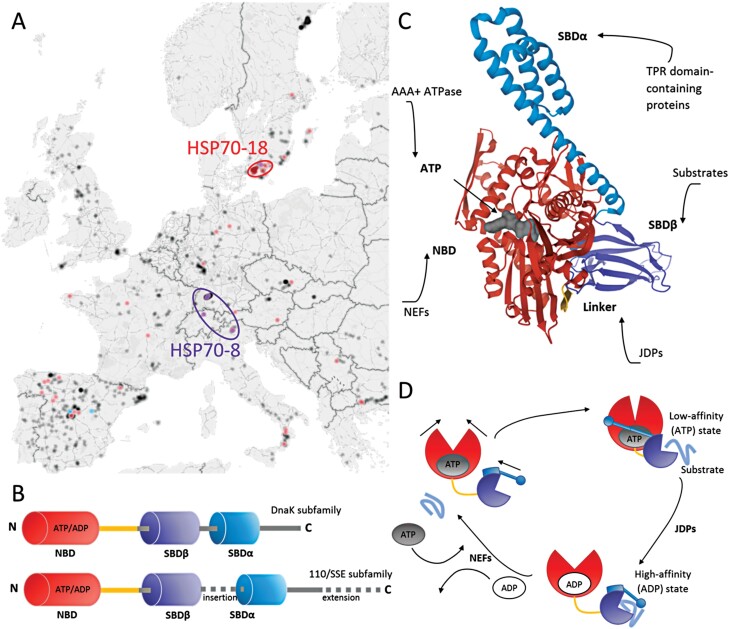
HSP70 sequence conservation, domain structure, and chaperone activity. (A) The frequency of mutations in *HSP70-8* and *HSP70-18* coincides with geographical location. The geographical distribution of all Arabidopsis accessions collected in the 1001 genome database (black) overlaid with the occurrence of high-impact mutations in *HSP70-8* (purple), *HSP70-13* (blue, no significant clustering), and *HSP70-18* (red). Data retrieved from the 1001 genome database (Weigel and Mott, 2009) and visualized using a Microsoft Excel 3D map. (B) Domain organization of DnaK and 110/SSE subfamilies (Lin *et al.*, 2001; Sarkar *et al.*, 2013). (C) Structure of HSP70 with bound ATP. Modeled using the crystal structure of bacterial DnaK (PDB ID: 4B9Q; Kityk *et al.*, 2012). Red, nucleotide-binding domain (NBD); blue, substrate-binding domain SBDα; violet, substrate-binding domain SBDβ; gray, ATP; yellow, linker. Sites of protein–protein interactions with HSP70 co-chaperones are indicated, including tetratricopeptide repeat (TPR) proteins, J-domain proteins (JDP), and nucleotide exchange factors (NEF). (D) Chaperone cycle of HSP70s. The interaction with JDPs stimulates ATP hydrolysis and the transition to the ADP-bound state with a high affinity for protein substrates. NEFs facilitate the HSP70 conversion back to the low-affinity state, the dissociation of ADP and rebinding of ATP, and the release of the protein substrate (Rosenzweig *et al.*, 2019).

HSP70s are highly conserved ATP-dependent chaperones, and this family shows the highest sequence similarity within a large evolutionary distance (Borchiellini *et al.*, 1998). An example is DnaK protein (prokaryotic HSP70), which shares ~50% amino acid identity with eukaryotic HSP70s. The genetic conservation of the HSP70 sequence is also evident in Arabidopsis accessions. The frequency of mutations is low, seems to correlate with geographical distribution (Fig. 1A), and 10 proteins show zero high-impact mutations (Table 1). Plant HSP70s share at least two of the four structural features of the archetype bacterial HSP70 DnaK: an N-terminal, 45 kDa nucleotide-binding domain (NBD), followed by a 15 kDa substrate-binding domain (SBDβ), a 10 kDa helical lid domain (SBDα), and a disordered C-terminal tail of variable length (Fig. 1C; Sarkar *et al.*, 2013). In eukaryotic cytosolic and nuclear HSP70s, the disordered C-terminal tail frequently ends with a characteristic charged motif (EEVD) that interacts with specific co-chaperones containing TPR repeats, including HSP-organizing protein (HOP; transfer of unfolded HSP70 substrates to foldases; Rosenzweig *et al.*, 2019), HSP-interacting protein (HIP; stabilization of the high-affinity ADP-bound state of HSP70; Zuiderweg *et al.*, 2017), and C-terminus of HSP70-interacting protein (CHIP; ubiquitination mediating degradation of unfolded HSP70 substrates via the proteasome; Fernández-Fernández *et al.*, 2017).

**Table 1.** Overview of the HSP70 family in the model plant Arabidopsis

UniProt	Protein names	AGI	SUBA/UniProt (∗) detected in extracellular space	Mutant phenotype	No. of gene models	High-impact mutations in 1001 genomes database
P22953	HSP70-1	AT5G02500	Cytosol, nucleus∗	A: altered plant growth and development (1), disabled immune response (2, 3), enhanced tolerance to heat shock, hypersensitive to ABA, compromised in the induced stomatal closure (2), γ-ray hypersensitivity and tolerance to salt, cadmium, and arsenic (4) B: did not reveal any difference in the resistance against pathogens *Hyaloperonospora parasitica* and *Pseudomonas syringae* pv *tomato* (3), more susceptible to *P. syringae *pv.* Maculicola* Δ*hopI* (5)	2	1 (SNPs)
Q9S7C0	HSP70-14	AT1G79930	Cytosol, nucleus	B: indistinguishable from the wild type (6)	2	4 (SNPs)
Q9SAB1	HSP70-16	AT1G11660	Cytosol, nucleus	B: failed flower opening, abnormal floral organ formation, impaired fertilization and seed setting (7), suppressed seed germination under cold stress conditions (8)	4	1 (SNPs)
P22954	HSP70-2	AT5G02490	Cytosol, nucleus	B: did not reveal any difference in the resistance against pathogens *Hyaloperonospora parasitica* and *Pseudomonas syringae* pv *tomato* (3), more susceptible to *P. syringae *pv.* Maculicola* Δ*hop* (5)	1	0
O65719	HSP70-3	AT3G09440	Cytosol, nucleus∗	B: did not reveal any difference in the resistance against pathogens *Hyaloperonospora parasitica* and *Pseudomonas syringae* pv *tomato* (3)	4	0
Q9LHA8	HSP70-4	AT3G12580	Cytosol, nucleus	C: abnormal embryogenesis, defective seedlings with high levels of ROS–RNAi in an hsc70-1 mutant background (9)	1	0
Q9S9N1	HSP70-5	AT1G16030	Cytosol∗	B: delayed growth under normal conditions, reduced survival of both seeds and seedlings after severe heat treatments, decreased growth activity under water deficit (10)	1	0
Q9SKY8	HSP70-8	AT2G32120	Cytosol		2	11 (SNPs)
F4JMJ1	HSP70-17	AT4G16660	Endoplasmic reticulum		2	3 (SNPs)
Q9LKR3	HSP70-11 (BIP1)	AT5G28540	Endoplasmic reticulum, nucleus∗	B: BIP1+BIP2 double mutation has an effect on the fusion of polar nuclei during female gametophyte development (11)	1	0
Q39043	HSP70-12 (BIP2)	AT5G42020	endoplasmic reticulum, nucleus ^∗^	B: compromised secretion of PR1 (12), enhanced fungal colonization (13), BIP1+ IP2 double mutation has an effect on the fusion of polar nuclei during female gametophyte development (11)	3	0
Q8H1B3	HSP70-13 (BIP3)	AT1G09080	Endoplasmic reticulum, nucleus	B: BIP1+BIP2+BIP3 triple mutation is pollen lethal (14)	2	5 (SNPs), 1 (INS), 1 (DEL)
F4HQD4	HSP70-15	AT1G79920	Golgi, cytosol, nucleus	B: severe growth retardation, impaired stomatal closure and accelerated wilting, enhanced tolerance to potyvirus infection (6)	4	0
Q9LDZ0	HSP70-10	AT5G09590	Mitochondrion∗		1	0
Q8GUM2	HSP70-9	AT4G37910	Mitochondrion, cytosol∗	B: severe growth defects, abnormal mitochondria and alterations to respiration because of an inhibition of the cytochrome *c* oxidase (15)	2	0
Q9C7X7	HSP70-18	AT1G56410	Plastid, cytosol∗		1	10 (SNPs), 7 (INS), 46 (DEL)
Q9STW6	HSP70-6	AT4G24280	Plastid∗	B: variegated cotyledons, malformed leaves, growth retardation, impaired root growth (16), *hsp70-6/hsp70-7* double mutants are lethal (17)	1	0
Q9LTX9	HSP70-7	AT5G49910	Plastid	B: *hsp70-6/hsp70-7* double-mutants are lethal (17), defective protein import into chloroplasts during early developmental stages (18)	1	1 (SNPs)

Based on UniProt (Bateman *et al.*, 2017), SUBA 4.0 (Hooper *et al.*, 2017), the 1001 genome database (Weigel and Mott, 2009), and the previously reported response of *HSP70* overexpression (A), loss-of-function mutation (B), and RNAi (C): 1, Sung and Guy (2003); 2, Clément *et al.* (2011); 3, Noël *et al*. (2007); 4, Cazalé *et al.* (2009); 5, Jelenska *et al.* (2010); 6, Jungkunz *et al.* (2011); 7, Chen *et al.* (2019); 8 Ashraf *et al.* (2021); 9, Lee *et al.* (2009); 10, Kozeko (2021); 11, Maruyama *et al.* (2010); 12, Wang *et al.* (2005); 13, Qiang *et al.* (2012); 14, Maruyama *et al.* (2014); 15, Wei *et al.* (2019); 16, Su and Li (2008); 17, Latijnhouwers *et al.* (2010); 18, Su and Li (2010). SNP, single nucleotide polymorphism; INS, insertion; DEL, deletion.

### Chaperone activity of HSP70s

The mediation of protein folding and translocation is the best-described function of HSP70s. The promiscuity of the HSP70 interaction and efficient targeting to substrate proteins are based on a transient interaction of the SBD domain (Fig. 1B–D) with a short degenerative sequence motif that is frequently found in virtually all proteins (Rüdiger *et al.*, 1997; Kityk *et al.*, 2012). The HSP70–substrate interaction depends on a complicated allosteric mechanism that couples ATP hydrolysis in the NBD with substrate capture by the SBD (described in more detail in Qi *et al.*, 2013; Kityk *et al.*, 2015; Rosenzweig *et al.*, 2019). The HSP70 binds to protein substrates with a significantly increased affinity in the ADP-bound state, and ATP hydrolysis is essential for its chaperone function (Kityk *et al.*, 2018). However, the intrinsic ATPase rate is very low (Kityk *et al.*, 2012) and requires co-chaperones, namely J-domain proteins (JDPs; accelerate ATP hydrolysis) and nucleotide exchange factors (NEFs; accelerate ADP–ATP exchange) (Abrams *et al.*, 2014; Bracher and Verghese, 2015; Zuiderweg *et al.*, 2017). The whole chaperone cycle is illustrated in Fig. 1D. In contrast to other chaperones, HSP70s do not require self-interaction for the chaperone functions (Takakuwa *et al.*, 2019).

Besides the described interactions and chaperone complexes that facilitate protein folding, HSP70s are essential for driving the movement of proteins across the membrane and protein translocation into the endoplasmic reticulum (ER), mitochondria, and plastids (Su *et al.*, 2010; Schleiff and Becker, 2011; Nakai, 2018; Sun *et al.*, 2021). Translocases have narrow channels, limiting the transport to a completely unfolded chain or, at most, α-helices (Craig, 2018). Interestingly, organellar HSP70s are more critical for the translocation process than their cytosolic counterparts, and form the core of the so-called import motors. There are different models for the HSP70 motor action mechanism that pulls the protein into an organelle (e.g. Sousa and Lafer, 2019), but the substrate–HSP70 interaction itself is identical to that of the standard chaperone cycle and requires JDP-driven ATP hydrolysis.

### HSP70s mediate quality control

HSP70s have an essential role in protein quality control. The fate of misfolded and aggregated proteins is decided by a combination of HSP70 and its interactors, and the defective protein is targeted for degradation through the ubiquitin–proteasome system, as well as through autophagy pathways. The most frequent means of protein degradation in eukaryotic cells requires the ubiquitin–26S proteasome system. Proteins are targeted via ubiquitination through the sequential action of three discrete enzymes: the ubiquitin-activating enzyme (E1), ubiquitin-conjugating enzyme (E2), and ubiquitin ligase (E3). The E3 ligase is responsible for substrate recognition, and a highly conserved and chaperone-dependent E3 ligase is CHIP. This enzyme interacts with HSP70 and ubiquitinates protein substrates captured by HSP70. CHIP has been extensively studied in many model organisms, including plants (reviewed in, for example, Zhang *et al.*, 2021), and accumulated knowledge suggests that it is an integral component of both biotic and abiotic stress control. Chaperone-mediated autophagy is mediated by cytosolic HSP70s and targets proteins containing the signal motif KFERQ (Fernández-Fernández *et al.*, 2017). This was the first described chaperone-assisted pathway but requires a receptor that seems to be missing in plant lineages (Lescat *et al.*, 2020). Similarly, the autophagy pathway mediated by endoplasmic HSP70 has not yet been confirmed in plants (Cha-Molstad *et al.*, 2015).

### Classification and localization of plant HSP70s

The plant HSP70 protein superfamily consists of two subfamilies: HSP70 (DnaK) and HSP110/SSE (Fig. 1B). The Arabidopsis genome encodes 14 and four members of HSP70 and HSP110/SSE subfamilies, respectively. HSP110/SSE exhibits homology to yeast SSE1 and human HSP110. The HSP110 sequence contains an insertion of acidic residues in the substrate-binding domain, an extension of the C-terminal domain, and the resulting protein is significantly larger (Fig. 1B).

All eukaryotes possess multiple HSP70s. In plants, four distinct sets function in the cytosol, mitochondria, chloroplasts, and endoplasmic reticulum (Table 1). This localization is determined by conserved N- and C-terminal sequences and is often used to divide HSP70s into four major subgroups: cytosolic/nuclear (EEVD motif), ER (HDEL motif), plastidic (PEGDVIDADFTDSK motif), and mitochondrial (PEAEYEEAKK motif) (Guy and Li, 1998). Those targeted to the same compartment share a similar evolutionary history. HSP70s targeted to the ER and cytosol resulted from gene duplication and subsequent divergence, and HSP70s targeted to mitochondria and chloroplasts evolved by gene transfer from endosymbionts and are more related to the prokaryotic DnaK (Waters and Rioflorido, 2007; Huang *et al.*, 2008)

### The low correlation between gene expression and protein abundance indicates extensive post-transcriptional or post-translational control of HSP70s

**Fig. 2.**
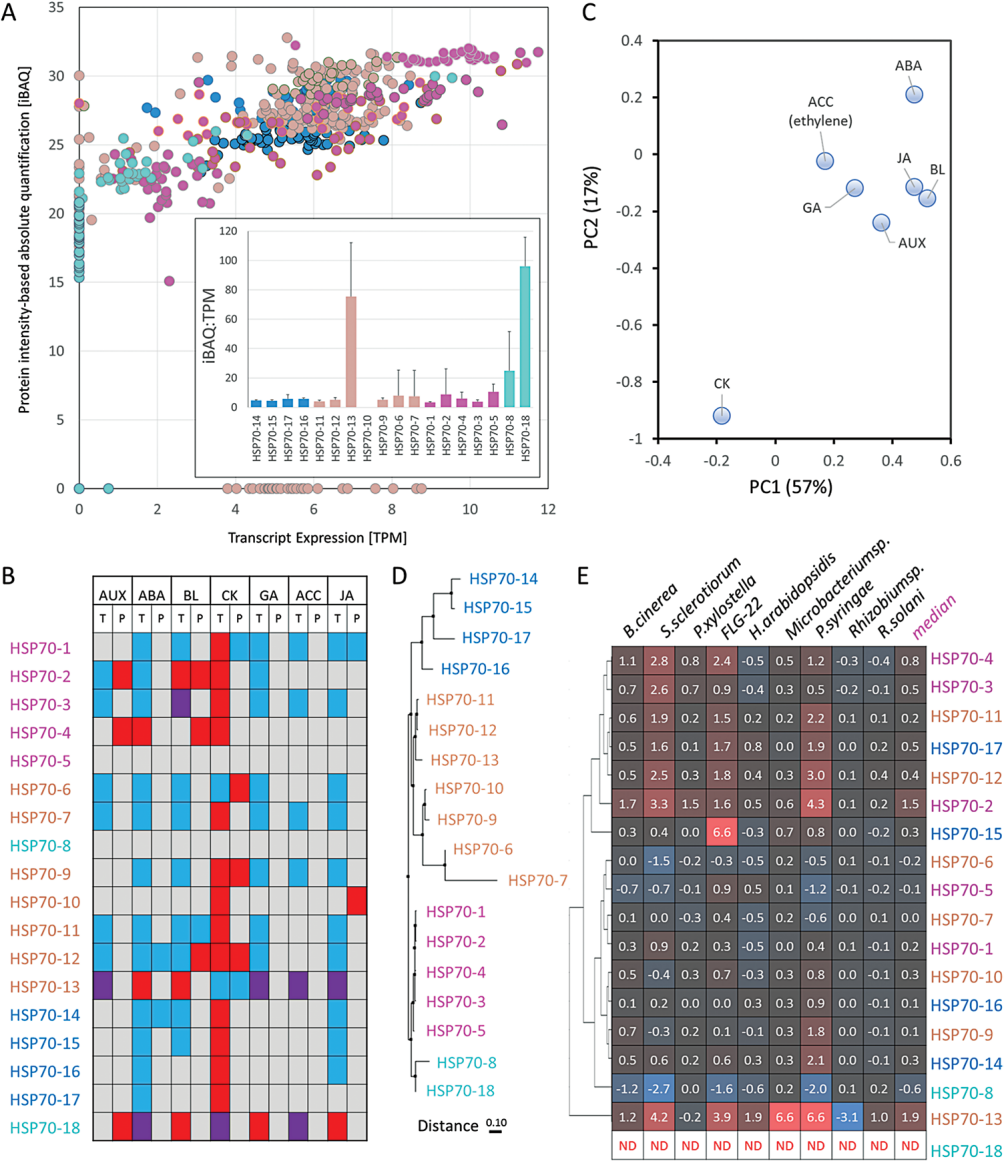
Transcriptional and translational control of HSP70. (A) Gene expression and estimated protein abundance of HSP70s in Arabidopsis. Comparison of available profiles across a set of 30 matching tissues from the Arabidopsis tissue atlas (Mergner *et al.*, 2020). The inset plot represents the average protein:transcript ratio with the SD. (B) Phytohormones regulate HSP70 biosynthesis. Simplified heat map visualization of reported transcriptional (T; ThaleMine; Krishnakumar *et al.*, 2017) and protein (P; Černý *et al.*, 2016) response to phytohormones. (C) Principal component analysis of the data in (B). Red, up-regulation/increase in protein abundance; blue, down-regulation, decrease in protein abundance; purple, mixed response; AUX, auxin; ABA, abscisic acid; BL, brassinosteroid; CK, cytokinin; GA, gibberellin; ACC, ethylene precursor; JA, jasmonate. (D) Sequence feature similarity visualized by Jalview (Waterhouse *et al.*, 2009) and (E) Arabidopsis *HSP70* genes in response to biotic interaction and flagellin peptide fragment (FLG-22; elicits defense response). The ratio represents the median log2 expression compared with mock-treated plants. Expression data were retrieved from the Arabidopsis RNA-Seq Database (Zhang *et al.*, 2020).

The analysis of available Arabidopsis protein data and expression profiles showed that only a minor fraction of HSP70s has a positive linear correlation between transcript and protein abundance (Fig. 2A). A higher correlation (Spearman correlation coefficient *r* >0.7) was found only for HSP70-4, HSP70-5, and HSP70-8. The highest deviations were found for HSP70-18 (expression data available only for imbibed seeds) and HSP70-10 (not found in the comparative dataset stored in the Arabidopsis tissue atlas). Translation elongation rates, post-transcriptional regulations, post-translational modifications, and protein life span impact the resulting protein abundances. Protein degradation rates have been estimated for seven Arabidopsis HSP70s (L. Li *et al.*, 2017) and show significant differences. The lowest average degradation rates were found for plastid-localized isoforms HSP70-6 (0.069 d^–1^) and HSP70-7 (0.082 d^–1^), indicating an intermediate protein turnover rate. In contrast, cytosolic HSP70s showed a rapid degradation, with an average degradation rate of HSP70-14 reaching 1.878 d^–1^. Information about the post-transcriptional control of plant HSP70s is missing, but it has been demonstrated that *HSP70* pseudogenes, including products of *HSP70* alternative splicing, may act as long non-coding RNAs and interfere with the HSP70 translation (Bernabò *et al.*, 2020). Arabidopsis *HSP70-18* was considered a pseudogene, but the proteome analyses showed that this is not the case (Fig. 2A). There are 35 predicted *HSP70* gene models, and only eight Arabidopsis *HSP70* genes are missing putative alternatively spliced variants (Table 1). It is possible that at least some of these variants could participate in transcriptional control, but the sequences are highly similar. Except for AT1G79920.2, the available peptide-based evidence cannot confirm or exclude the existence of the corresponding proteins (van Wijk *et al.*, 2021, Preprint).

### Plant hormones control *HSP70* expression

The phytohormones are small-molecule regulators that collectively regulate plant growth and development, and facilitate the integration of environmental cues, including biotic and abiotic stressors. It is thus not surprising to find Arabidopsis HSP70 among the list of phytohormone-responsive genes and proteins (Fig. 2B, C). Only two cytosolic HSP70s appear to be missing in response to phytohormones, namely HSP70-5 and HSP70-8. Somewhat counterintuitive is the reported response to hormones traditionally associated with a stress response. Abscisic acid, jasmonates, and ethylene showed a predominantly negative effect on *HSP70* expression and the abundances of the corresponding proteins. Arabidopsis data for salicylic acid treatment were not available in the selected datasets, but previous proteomic analyses showed a decrease in HSP70 after salicylic acid treatment of *Cucumis sativus* and *Cunninghaimia lanceolata* (Černý *et al.*, 2016). In contrast, cytokinin up-regulates most HSP70s and positively impacts HSP70 abundances (Fig. 2B). That could coincide with the predicted role of cytokinin in response to heat shock (Černý *et al.*, 2014). Interestingly, abscisic acid and cytokinin antagonistically regulate many developmental processes (Huang *et al.*, 2018), and the observed antagonistic effect on HSP70s (Fig. 2B, C) indicates that its regulation could be a part of the crosstalk between these two phytohormones.

### HSP70s are subject to extensive post-translational control

**Fig. 3.**
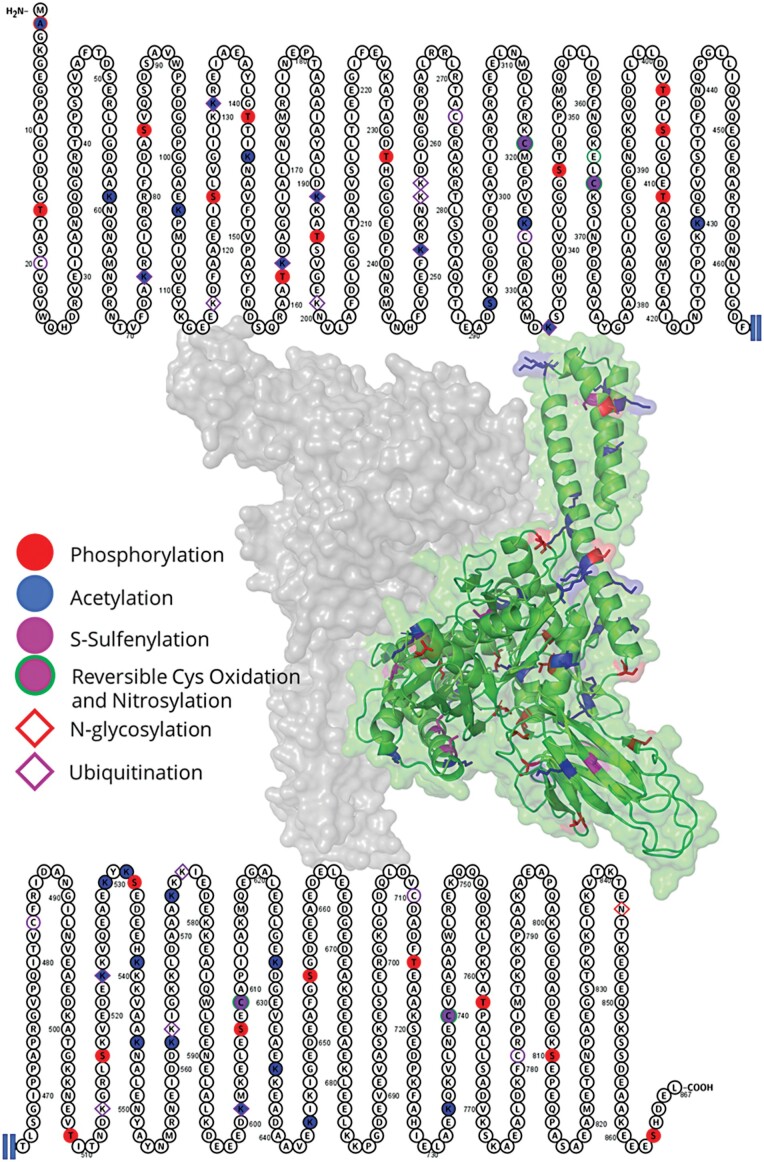
Reported post-translational modifications of Arabidopsis HSP70s mapped onto the consensus sequence. The consensus of the HSP70 sequence generated by Jalview (Waterhouse *et al.*, 2009), visualized by Protter (Omasits *et al.*, 2014), and projected into the 3D dimeric structure by SWISS-MODEL (Waterhouse *et al.*, 2018). Data were retrieved from the Plant PTM Viewer database (Willems *et al.*, 2019).

The Plant PTM viewer lists almost 300 HSP70 peptides with a post-translational modification (Willems *et al.*, 2019). The most frequent modifications are phosphorylation (65 peptides), acetylation (53 peptides), sulfenylation (33 peptides), and ubiquitination (27 peptides). However, these are the most intensively studied plant protein modifications, and the apparent enrichment probably does not reflect the true proportion of different modifications. As illustrated in Fig. 3, the sites of these modifications are highly conserved and most can be mapped onto the consensus of HSP70 family sequences. Experimental evidence in plant protein science is largely missing, but HSP70 modifications have been established as the so-called ‘chaperone code’ and can determine protein localization, selectivity, activity, and protein–protein interactions (Cloutier and Coulombe, 2013; Nitika *et al.*, 2020). These modifications represent both the fine-tuning of HSP70 biology and a means of rapid response to stress (Xu *et al.*, 2019). The activation of *HSP70* expression is rapid, but corresponding protein biosynthesis is significantly slower. For instance, the delay in heat-induced accumulation of Arabidopsis HSP70-4 and HSP70-5 is >2 h compared with the corresponding transcripts (Palmblad *et al.*, 2008). Post-translational modifications are thus the first line of defense. Phosphorylation and acetylation have been shown to regulate the oligomerization of HSP70s (Morgner *et al.*, 2015). The dimerization of HSP70s effectively buries its binding domains and inhibits protein–protein interactions (Takakuwa *et al.*, 2019), and thus the regulation of the monomer/oligomer pool represents a rapid control mechanism to regulate the activity of HSP70s.

The Arabidopsis HSP70 sequences contain on average 1.2% cysteines, and sulfenylation and nitrosylation have been reported for these residues (Fig. 3). Cysteine is the most reactive proteinogenic amino acid, and thiols often act as a redox molecular switch regulating protein activity (e.g. Černý *et al.*, 2013). In fact, sulfenylation of HSP70 has been demonstrated to activate heat shock transcription and induce thermotolerance in yeast (Wang *et al.*, 2012). Interestingly, HSP70 may also directly participate in reactive oxygen metabolism by regulating the transport of superoxide dismutase into mitochondria (Zemanovic *et al.*, 2018).

### Role of HSP70s in plant immunity

Analysis of publicly available data stored in the Arabidopsis RNA-Seq Database (Zhang *et al.*, 2020) showed that most *HSP70* genes are up-regulated in response to biotic stressors (Fig. 2E). Only cytosolic *HSP70-8* and chloroplastic *HSP70-6* and *HSP70-7* were predominantly repressed, and expression profiles for HSP70-18 were unavailable. Comparison of the expression profile and sequence feature similarity (Fig. 2D, E) did not show any significant similarity in the clusterings (adjusted Rand index 0.16), indicating that the response to biotic stress is not correlated with the HSP70 sequence. However, the contrasting response of chloroplastic *HSP70* genes and the available literature indicate that HSP70 localization is the key factor.

**Fig. 4.**
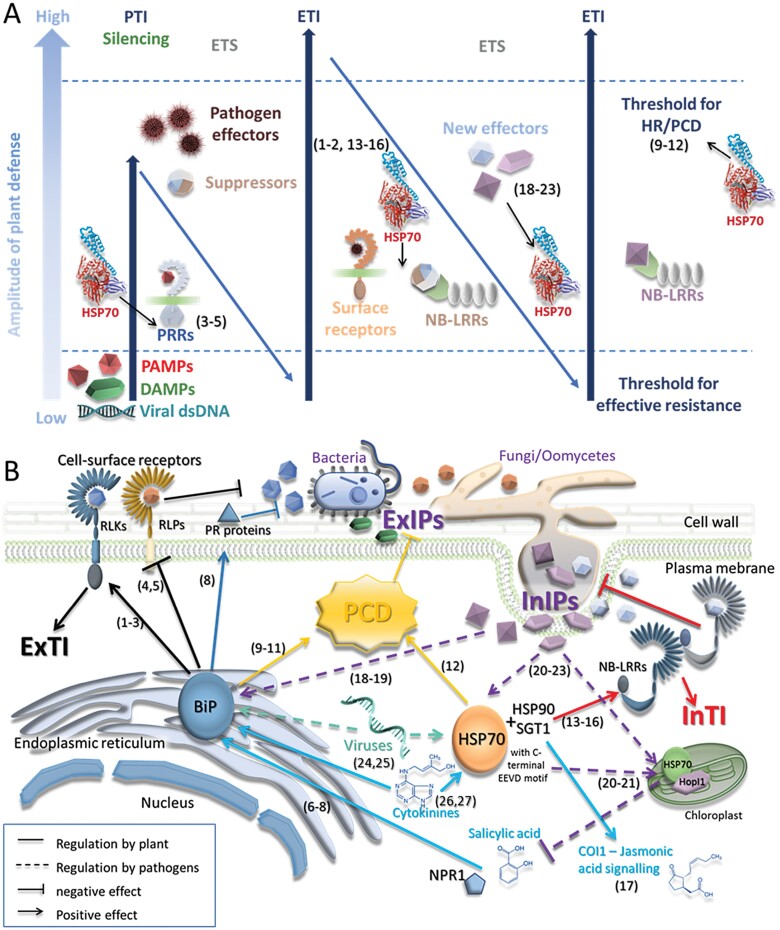
HSP70s and plant immunity. (A) Simplified diagram representing the classical view of plant–pathogen interaction, with the highlighted role for HSP70s in pattern-triggered immunity (PTI), effector-triggered immunity (ETI), programmed cell death, and in interaction with pathogen effector proteins. PAMPs, pathogen-associated molecular patterns; DAMPs, damage-associated molecular patterns; PPRs, pattern recognition receptors; NB-LRRs, nucleotide-binding site-leucine-rich repeats; HR, hypersensitive response; PCD, programmed cell death; ETS, effector-triggered susceptibility. (B) Subcellular localization and interactions of HSP70 in the spatial immunity model. BiPs are components of extracellularly triggered immunity (ExTI). These HSP70s are regulated by salicylic acid and NPR1, and play a role in the secretion of pathogenesis-related (PR) proteins. The cytosolic HSP70s participate in intracellularly triggered immunity (InTI) and influence perception of jasmonic acid. Cytokinin up-regulates most *HSP70* genes and positively impacts HSP70 abundance. Moreover, HSP70s interact with pathogen effector proteins and are involved in virus replication and translocation. COI1, coronatine-insensitive protein 1; ExIPs, extracellular immunogenic patterns; InIPs, intracellular immunogenic patterns; NB-LRRs, nucleotide-binding site-leucine-rich repeats; NPR1, non-expresser of PR genes 1; PCD, programmed cell death; RLKs, receptor-like kinases; RLPs, receptor-like proteins. References: 1–2, Liebrand et al. (2012, 2014); 3, Nekrasov *et al.* (2009); 4–5, Park et al. (2010, 2014); 6, Nagashima *et al.* (2014); 7, Poór *et al.* (2019); 8, Wang *et al.* (2005); 9, Carvalho *et al.* (2014); 10, Qiang *et al.* (2012); 11, Xu *et al.* (2012); 12, Kanzaki *et al.* (2003); 13, Noël *et al*. (2007); 14, Thao *et al.* (2007); 15, Nakashima *et al.* (2008); 16, Shirasu (2009); 17, X.-C. Zhang *et al.* (2015); 18, Jing *et al.* (2016); 19, Zhou *et al.* (2021); 20–21, Jelenska et al. (2007, 2010); 22, Lee *et al.* (2018); 23, Kim and Hwang (2015); 24, Hýsková *et al.* (2021b); 25, Ye *et al.* (2011); 26, Černý *et al*. (2016); 27, Krishnakumar *et al.* (2017). For details about the spatial immunity model, see, for example, van der Burgh *et al*. (2019).

Plants respond to pathogen invasion using a two-branched innate immune system consisting of PAMP (pathogen-associated molecular pattern)-triggered immunity (PTI) and effector-triggered immunity (ETI; Abdul Malik *et al.*, 2020). PTI represents an early response mechanism mediated by pattern recognition receptors (PRRs) that recognize and bind PAMPs (Nürnberger and Kemmerling, 2009). ETI is a reaction to pathogen-secreted effectors interfering with PTI. This second defensive barrier recruits resistance (R) proteins that specifically recognize pathogen effectors and usually triggers a hypersensitive response and cell death (Abdul Malik *et al.*, 2020). HSP70s are an integral part of plant immunity and participate in both PTI and ETI responses (Fig. 4).

### Cytosolic HSP70s regulate ETI

The HSP70 subfamily (DnaK) members with a C-terminal EEVD motif (sometimes referred to as heat shock cognate 70 chaperones) regulate immune responses in plants by cooperating with cytosolic chaperones such as SGT1, HSP90, and RAR1. This interaction is an integral part of the processing and folding of NB-LRR (nucleotide-binding domain-leucine-rich repeat) immune sensors (Shirasu, 2009). There are four cognate *HSP7*0 genes in Arabidopsis (isoforms 1–4), and three of these are strongly up-regulated in response to different pathogens (Fig. 2E). Limited evidence indicates that these *HSP70* genes are at least partially redundant, and experiments with a loss-of-function mutation of individual *HSP70* isoforms 1, 2, and 3 did not reveal any difference in the resistance against pathogens (Noël *et al*., 2007; Table 1). Surprisingly, *HSP70-1* is mostly unresponsive in biotic stress experiments (Fig. 2E; Table 2). A transgenic line overexpressing *HSP70-1* has a reduced NB-LRR-dependent immunity response (Noël *et al.*, 2007), but that could coincide with an attenuated expression of five different *HSP70* genes (Sung and Guy, 2003). The HSP70 interactor SGT1 is a co-chaperone of HSP90 and, in addition to ETI, also regulates auxin and jasmonic acid signaling by maintaining steady-state levels of the corresponding receptors TIR1 and COI1 (X.-C. Zhang *et al.*, 2015; Wang *et al.*, 2016). It has been proven that the COI1 stabilization is facilitated by SGT1b–HSP70–HSP90 chaperone complex (X.-C. Zhang *et al.*, 2015), and HSP70s thus directly influence hormonal perception.

**Table 2.** Summary of recent reports supporting the role of HSP70 in biotic interactions

Host	Pathogen	Arabidopsis ortholog	Regulation	Reference
*Arabidopsis thaliana*	*Phytophthora capsici*	HSP70-4	↑	(1)
		HSP70-5	↑	(1)
	*Pseudomonas syringae* pv *tomato*	HSP70-2	↑	(2)
		HSP70-4	↑	(2)
*Brassica rapa*	*Turnip mosaic virus*	HSP70-4	↑	(3)
		HSP70-1	↑	(3)
*Capsicum annum*	*Bemisia tabaci*	HSP70-2	↑	(4)
		HSP70-1	↑	(4)
	*Pepper golden mosaic virus*	HSP70-4	↑	(5)
*Castanea sativa*	*Phytophthora cinnamomi*	HSP70-1	↑	(6)
		HSP70-4	↓	(6)
		HSP70-12	↑	(6)
		HSP70-10	↑↓	(6)
		HSP70-16	↑	(6)
*Cicer arietinum*	*Fusarium oxysporum*	HSP70-1	↑	(7)
*Glycine max*	*Heterodera glycines*	HSP70-2	↑	(8)
		HSP70-4	↑	(8)
*Hordeum vulgare*	*Phytophthora palmivora*	HSP70-4	↓	(9)
	*Blumeria graminis*	HSP70-4	↑	(10)
	*Puccinia graminis*	HSP70-1	↑	(11)
*Malus domestica*	*Alternaria alternata*	HSP70-4	↑↓	(12)
		HSP70-5	↑	(12)
*Nicotiana benthamiana*	*Potato virus X*	HSP70-12	↑	(13)
	*Potato virus Y*	HSP70-4	↑	(14)
	*Tobacco mosaic virus*	N/A	↑	(15)
*Nicotiana tabacum*	*Potato virus Y*	N/A	↑	(16)
*Pennisetum glaucum*	*Puccinia substriata*	HSP70-4	↓	(17)
*Pisum sativum*	*Pea seed-borne mosaic virus*	HSP70-3	↑	(18)
*Quercus suber*	*Phytophthora cinnamomi*	HSP70-11	↑	(19)
*Saccharum spp.*	*Sporisorium scitamineum*	HSP70-2	↓	(20)
*Solanum lycopersicum*	*Fusarium oxysporum f. sp. radicis-lycopersici*	HSP70-10	↑	(21)
	*Phytophthora parasitica*	HSP70-4	↑	(22)
	*Tomato yellow leaf curl virus*	N/A	↓	(23)
		HSP70-4	↓	(24)
		HSP70-2	↓	(24)
*Solanum lycopersicum*	*Rhizopus nigricans*	HSP70-7	↓	(25)
*Solanum tuberosum* L.	*Synchytrium endobioticum*	HSP70-4	↓∗	(26)
		HSP70-10	↓∗	(26)
		HSP70-2	↑∗	(26)
*Triticum aestivum*	*Blumeria graminis*	HSP70-2	↑	(27)
	*Puccinia striiformis*	Hsp70-2	↑	(27)
	*Chinese wheat mosaic furovirus*	HSP70-4	↑	(28)
*Vigna unguiculata*	*Cowpea severe mosaic virus*	HSP70-2	↓	(29)
		HSP70-3	↓	(30)
		HSP70-4	↓	(30)
		HSP70-5	↓	(30)
		HSP70-9	↓	(30)
		HSP70-18	↓	(30)
*Vitis vinifera*	*Plasmopara viticola*	HSP70-4	↑	(31)
*Zea mays*	*Sugarcane mosaic virus*	HSP70-12	↑	(32)

For the sake of simplicity, diverse non-canonical nomenclature has been replaced with the corresponding Arabidopsis orthologs. Asterisks indicate differences between susceptible and resistant cultivars. References: 1, Song *et al.* (2015); 2, Noël *et al*. (2007); 3, Lyu *et al.* (2020); 4, Wu *et al.* (2019); 5, Góngora-Castillo *et al.* (2012); 6, Saiz-Fernández *et al.* (2020); 7, Gupta *et al.* (2017); 8, Klink *et al.* (2007); 9, Berka *et al.* (2020); 10, Molitor *et al.* (2011); 11, Sharma Poudel *et al.* (2019); 12, C. Zhang *et al.* (2015); 13, Ye *et al.* (2011); 14, Hafrén *et al.* (2010); 15, Caplan *et al.* (2009); 16, Hýsková *et al.* (2021b); 17, Crampton *et al.* (2009); 18, Cerna *et al.* (2017); 19, Coelho *et al.* (2021); 20, Singh *et al.* (2019); 21, Vitale *et al.* (2014); 22, Naveed and Ali (2018); 23, Gorovits *et al.* (2013); 24, Chen *et al.* (2013); 25, Pan *et al.* (2013); 26, Szajko *et al.* (2020); 27, Guo *et al.* (2021); 28, Yang *et al.* (2017); 29, Paiva *et al.* (2016); 30, Varela *et al.* (2017); 31, Liu *et al.* (2021); 32, Chen *et al.* (2017).

The critical role of cytosolic HSP70s in plant defense is supported by the fact that these proteins are targeted by pathogen effector proteins. HopI1, a virulence effector of pathogenic *Pseudomonas syringae*, binds directly to the host HSP70 and recruits it to chloroplasts, the site of HopI1 localization (Jelenska et al., 2007, 2010). The interaction was confirmed by co-immunoprecipitation for all four Arabidopsis isoforms (1–4) and for chloroplastic HSP70-6. Further, plants with depleted cytosolic HSP70-1 were more susceptible to *P. syringae* (Jelenska *et al.*, 2010), and silencing of cytosolic HSP70 in pepper (*Capsicum annuum*) increased the plant’s susceptibility to *Xanthomonas campestris* (Kim and Hwang, 2015). Finally, the effector Pi23226 of *Phytophthora infestans* was co-immunoprecipitated with HSP70s, and transient overexpression of these HSP70s in *Nicotiana benthamiana* inhibited *Phytophthora* growth (Lee *et al.*, 2018).

### HSP70s resident in the endoplasmic reticulum are related to endoplasmic reticulum-mediated plant immunity

Recent studies have pointed out the ability of plant pathogens to utilize their effectors to bind to the host ER stress pathway and manipulate it to their advantage (reviewed, for example, in Jing and Wang, 2020). ER stress occurs when the ER’s ability to fold proteins becomes saturated and can induce programmed cell death (PCD; Eichmann and Schäfer, 2012). Cells have evolved an unfolded protein response mechanism that induces the expression of genes that encode ER chaperones, including *HSP70* genes. ER-resident HSP70s (also called binding protein, BiPs) participate in plant immunity as a central regulator and are the link to ER-supported immunity functions. *BiP* genes are induced by salicylic acid (Nagashima *et al.*, 2014; Poór *et al.*, 2019) and multiple biotic stressors (Figs 2E, 4B), and the expression is reportedly regulated by NPR1, the master regulator of systemic acquired resistance (Wang *et al.*, 2005). BiPs stabilize folding intermediates, prevent aggregation, and aid in the subsequent protein folding and assembly (Padmanabhan and Dinesh-Kumar, 2010). In contrast to mammals and yeast, flowering plants contain several BiP proteins (HSP11–13 in Arabidopsis; Noh *et al.*, 2003). BiP1 and BiP2 are highly similar based on their amino acid sequence, and they are ubiquitously expressed under non-stressed conditions, whereas BiP3 is more distantly related, highly induced upon ER stress, and therefore classified as a marker gene of the Arabidopsis unfolded protein response (Strasser, 2018). Several studies have shown that a process called ER quality control is essential for the proper function of the pattern recognition receptors, and part of this pathway relies on the BiP chaperone complex (Anelli and Sitia, 2008; Strasser, 2018). Nekrasov *et al.* (2009) reported that a subset of LRR-receptor-like kinases (RLKs), including EF-Tu (elongation factor thermounstable) receptor (EFR), require one or several ER complexes comprising SDF2, ERdj3B (Hsp40 co-chaperone), and BiP for their accumulation and subcellular localization. This chaperone complex regulates the biogenesis of multiple components of the plant defense system, including Cf proteins (resistance to the foliar pathogen *Cladosporium fulvum*; Liebrand *et al.*, 2012) and Ve1 (resistance to the vascular fungal pathogen *Verticillium dahliae*; Liebrand *et al.*, 2014). In contrast to the positive effect of cytosolic *HSP70* overexpression, BiP3 overexpression compromised immunity against *Xanthomonas oryzae* (Park et al., 2010, 2014). OsBiP3 can bind to the critical XA21 receptor that facilitates resistance, and the presence of high levels of BiP reduces its stability and function (Park *et al.*, 2010). The silencing of the individual BiPs did not affect EFR or Cf function, but a knockdown of the different BiPs in tomatoes resulted in a compromised Ve1-mediated resistance to *V. dahliae* (Nekrasov *et al.*, 2009; Liebrand et al., 2012, 2014). The *bip2* mutant showed compromised secretion of PR1, leading to an impaired resistance against the bacterial pathogen *Pseudomonas syringae* (Wang *et al.*, 2005).

HSP70s were found to play a role in developmentally regulated PCD (Rowarth *et al.*, 2020), and several studies suggest that BiPs are responsible for that (see, for example, Zhang *et al.*, 2014). Furthermore, this mechanism is utilized by both mutualistic fungi and pathogens. For example, the Arabidopsis mutant *bip2* showed enhanced fungal colonization rates compared with the wild type, and BiP protein levels were reduced in colonized wild-type roots compared with non-colonized roots, suggesting that impaired BiP accumulation supports fungal development during cell death-associated colonization (Qiang *et al.*, 2012). Similarly, silencing of *BiP* resulted in a delay of the hypersensitive response and PCD induced by *Xanthomonas oryzae* pv. *oryzae* (Xu *et al.*, 2012), and BiP-overexpressing lines displayed an accelerated hypersensitive response triggered by *Pseudomonas syringae* pv. *tomato* in soybean and tobacco (Carvalho *et al.*, 2014). It has also been found that the effector PsAvh262 secreted by *Phytophthora sojae* stabilizes BiPs and suppresses ER stress-triggered cell death (Jing *et al.*, 2016). Viruses can also exploit this pathway (Gaguancela *et al.*, 2016), and up-regulation of *BiP* was found in many previous studies, including response to *Potato virus X*, *Turnip crinkle virus*, *Garlic virus X*, and *Sugarcane mosaic virus* (Ye *et al.*, 2011; Lu *et al.*, 2016; Moon *et al.*, 2016; Chen *et al.*, 2017). A recent study showed that the BiP accumulation inhibits the ER to nucleus translocation of Bcl-2-associated athanogene (BAG7; Zhou *et al.*, 2021). This protein is a member of a multifunctional group of co-chaperones, and its role in heat tolerance, unfolded protein response, and basal immunity is well established (Williams *et al.*, 2010; Li *et al*., 2016; Y. Li *et al*., 2017). The accumulated evidence indicates that the BAG7 translocation to the nucleus is critical for its protective function and that its BiP-induced retention in the ER promotes susceptibility to pathogens (Zhou *et al.*, 2021).

### HSP70s mediate virus replication and cell to cell transport

*HSP70* genes have been found to be up-regulated in response to many viruses, and this effect is not limited to BiPs. For example, up-regulation or an increase in HSP70 was reported in response to *Tobacco mosaic virus* (*N. benthamiana*; cytosolic *HSP70* genes; Caplan *et al.*, 2009), *Turnip mosaic virus* (*Brassica rapa*; two cytosolic *HSP70* genes; Lyu *et al.*, 2020), and *Pea seed-borne mosaic virus* (*Pisum sativum*; two cytosolic *HSP70* genes; Cerna *et al.*, 2017). The role of these regulations is far from fully understood. The induction of *HSP70* may represent a host reaction to the synthesis of a large number of exogenous proteins (Alfenas-Zerbini *et al.*, 2009), and some resistance genes reportedly encode regulators of *HSP70* expression (Hayano-Saito and Hayashi, 2020). However, accumulated evidence indicates that the *HSP70* expression is, in fact, triggered by pathogens. Up-regulation of host *HSP70* genes has not been found in response to *Grapevine leafroll virus 3 closterovirus* infection, but this virus has its own *HSP70* gene (Espinoza *et al.*, 2007; Prator *et al.*, 2020). Aside from closteroviruses, plant viral genomes do not encode *HSP70* genes, but many viruses seem to recruit host HSP70s to assist in virion assembly, replication, and cell to cell movement (e.g. Mine *et al.*, 2012).

The cell to cell transport of plant viruses is mediated by specific virally encoded factors termed movement proteins and coat proteins which can create complexes with HSP70s (Nelson and Citovsky, 2005). It is believed that these complexes mediate virus interaction with the cytoskeleton and facilitate its transfer to and through the plasmodesmata (Aoki *et al.*, 2002; Krenz *et al.*, 2010). The interaction of HSP70s with coat proteins also facilitated the intracellular movement of *Tomato yellow leaf curl virus* into the nucleus, and the inactivation of HSP70 resulted in a decrease in viral replication (Gorovits *et al.*, 2013). Similarly, a positive effect of HSP70s on viral replication was reported for *Chinese wheat mosaic furovirus* (Yang *et al.*, 2017) and *Cowpea severe mosaic virus* (Paiva *et al.*, 2016; Varela *et al.*, 2017), and indicated for *Tomato bushy stunt virus* (Pogany *et al.*, 2008). Further, *Nicotiana tabacum* plants exposed to heat shock and inoculated with *Potato virus Y* showed higher amounts of HSP70 genes and higher virus content than the corresponding controls inoculated at standard temperature (Hýsková *et al.*, 2021b).

### HSP70 accumulation and plant resistance to pathogens

**Fig. 5.**
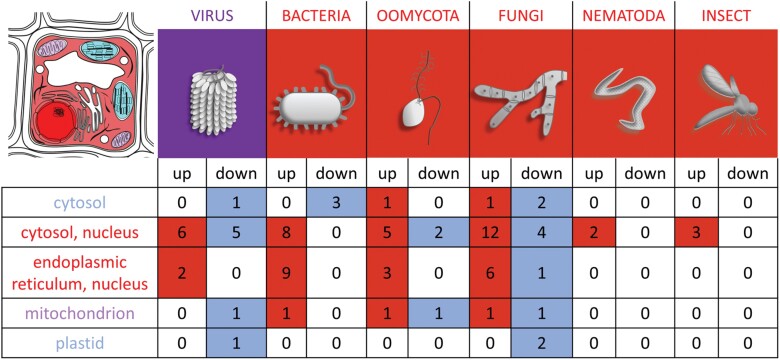
Comparison of the HSP70 response to biotic stress and its localization. Summary of reported transcriptomic and protein analyses outlined in Fig. 4B and Table 2. Localization is based on the expected localization of Arabidopsis HSP70s and corresponding putative Arabidopsis orthologs. The color of the cellular compartments represents the predominant response of the resident HSP70s to biotic stimuli. Red, blue, and purple represent a positive, negative, and mixed response to biotic stimuli, respectively.

The reported changes in *HSP70* expression and protein accumulation show a predominantly positive response to biotic stimuli (Fig. 5; Table 2), and many reports have indicated that an increase in the plant pathogen resistance positively correlates with the HSP70 accumulation. For instance, the comparison of tomato accessions showed that the accession resistant to *Phytophthora parasitica* had a significantly higher *HSP70* expression (Naveed *et al.*, 2018). Similarly, faster induction of *HSP70* genes was correlated with enhanced resistance to *Blumeria graminis* in barley (Molitor *et al.*, 2011). In contrast, at least some pathogens exploit the host’s HSP70 machinery, and a loss-of-function mutation or silencing of *HSP70* reportedly stimulate the resistance of the plant to viruses (reviewed in, for example, Hýsková *et al.*, 2021a). However, the effect of mutation is not that simple in a partially redundant *HSP70* gene family, and the interpretation should be carefully evaluated. The analysis of gene expression of Arabidopsis *hsp70-15* knockout plants showed that the mutation significantly increased *HSP70-4* (14-fold change) and *HSP70-2* (6-fold change) (Jungkunz *et al.*, 2011). These members of the DnaK subfamily are the most frequently found HSP70s in the biotic response, and the observed mutant resistance to the *Turnip mosaic virus* could coincide with that.

## Conclusion and future perspectives

Plant HSP70 research is lagging behind its animal counterparts, and our knowledge about post-transcriptional and post-translational control is only rudimentary. It is obvious that this should be the next objective in plant HSP70 research. Animal HSP70s play a significant signaling role in the extracellular space, particularly in the inflammatory and immune responses (see, for example, Mambula *et al.*, 2007). According to available data and similar to its animal counterparts, none of the Arabidopsis HSP70s contains a targeting signal for secretion. Despite the presence of cell walls, plants can secrete proteins without the consensus N-terminal peptide sequence via alternative, unconventional protein secretory pathways into the extracellular space. The exact extent of these alternative pathways in plants has not yet been fully elucidated, and the role of plant HSP70s in the extracellular space (if any) is unknown. However, at least nine Arabidopsis HSP70s have been detected in the extracellular space (Table 1), and it has been confirmed that the extracellular vesicles in the plant secretome contribute to the plant defense system (Regente *et al.*, 2017). The role of extracellular HSP70 could be evolutionarily conserved and could provide an additional explanation for the observed accumulation in response to pathogens. Finally, our review highlighted a prominent role for the plant hormone cytokinin in the regulation of *HSP70* genes. Cytokinin promotes plant resistance against pathogens such as bacteria, fungi, and pest insects (Akhtar *et al.*, 2020), and the up-regulation of *HSP70* genes is probably an overlooked part of that effect.
